# Studies on Mathematical Models of Wet Adhesion and Lifetime Prediction of Organic Coating/Steel by Grey System Theory

**DOI:** 10.3390/ma10070715

**Published:** 2017-06-28

**Authors:** Fandi Meng, Ying Liu, Li Liu, Ying Li, Fuhui Wang

**Affiliations:** 1 Institute of Metal Research, Chinese Academy of Sciences, Wencui Road 62, Shenyang 110016, Liaoning, China; fdmeng@imr.ac.cn (F.M.); liuying@imr.ac.cn (Y.Liu); liying@imr.ac.cn (Y.Li); fhwang@imr.ac.cn (F.W.); 2 Key Laboratory for Anisotropy and Texture of Materials (MoE), School of Materials Science and Engineering, Northeastern University, Wenhua Road 3-11, Shenyang 110819, Liaoning, China

**Keywords:** organic coatings, grey system theory, marine hydrostatic pressure, wet adhesion, lifetime prediction

## Abstract

A rapid degradation of wet adhesion is the key factor controlling coating lifetime, for the organic coatings under marine hydrostatic pressure. The mathematical models of wet adhesion have been studied by Grey System Theory (GST). Grey models (GM) (1, 1) of epoxy varnish (EV) coating/steel and epoxy glass flake (EGF) coating/steel have been established, and a lifetime prediction formula has been proposed on the basis of these models. The precision assessments indicate that the established models are accurate, and the prediction formula is capable of making precise lifetime forecasting of the coatings.

## 1. Introduction

Organic coatings are widely utilized to protect metals from corrosion and inevitably suffer degradation due to the exposure to the corrosive environment [1,2]. The lifetime prediction of the coatings, therefore, has realistic instructive significance in coating maintenance as well as the performance evaluation. However, lifetime prediction has always been a difficult task in the field of coating research [3], which could be mainly attributed to the complexity of the failure mechanisms of coatings. Moreover, for a variety of organic coatings applied in different environments, there is barely a universal model of lifetime prediction. Doherty et al. [4] established a mathematical model of blister area of the coating. The service life limit could be defined according to the critical area of blisters. Cmaitland and Mayne [5] put forward a prediction formula based on the electrolytic resistance of polymer films. Bierwagen et al. [6] developed a method for service time prediction from the electrochemical impedance spectroscopy (EIS) data. Shevchuk et al. [7] introduced a prediction model based on the physicochemical and electrochemical processes of the coating system. In general, a good reliability for the lifetime prediction models should be based on three premises: (i) the failure mechanism under a certain service environment is clear before the study of lifetime prediction; (ii) the experimental conditions follow the characteristics of the practical environment; (iii) the prediction results match the experimental results. However, most of the proposed prediction methods have not taken into account all of the premises, which might put the applicability of prediction models into question.

In related previous work [8,9], our group has studied the failure mechanisms of organic coatings in the simulated deep sea environment. The results revealed that marine hydrostatic pressure decreased the properties of coatings and accelerated the failure process. More importantly, the rapid loss of wet adhesion became the immediate cause for coating failure and thus the key factor controlling coating lifetime. The model of lifetime prediction in the deep sea environment, deservedly, should be established based on the analysis of adhesion. 

The adhesion of coating/metal system is a critical index for evaluating the coating performance [10]. If the adhesion fails, the other protection mechanisms will become worthless [11]. In particular, when the coatings are applied to the water soaking environment or high relative humidity (more than 60%) environment, the dry adhesion will change to wet adhesion, which is an important parameter of coating degradation [12]. The main difficulty in studying wet adhesion is the complexity of the de-adhesion process, which is influenced by numerous uncertain factors, including the transportation of water, interfacial electrochemical reactions, the surface state of the steel, the formation of blister and cathodic disbondment. These factors may interact with each other, making the degradation of wet adhesion more complicated [12,13,14,15,16]. It is for these reasons that few quantitative theories have been reported, not to mention the mathematical models and prediction equations.

The Grey System Theory (GST), established by Deng in 1982 [17], has been widely applied in many fields since its birth, such as economy, meteorology and engineering [18,19,20,21,22,23]. GST aims at the ambiguous systems which have unclear structures or unexplained principles. There are other modern mathematical methods involved in the study of ambiguous systems, such as “Black-Box” Method [24,25], Fuzzy System Theory [26,27], and Artificial Neural Network [28,29,30]. However, GST has its own features: suitable for a system with many uncertain reasons and a certain result [18,19]. For wet adhesion of the organic coating/steel system, there are many complex affecting factors during the de-adhesion process, but the certain measurements can be obtained. Consequently, GST is the preferred method for studying the models of wet adhesion.

In this study, variations of the wet adhesion of epoxy varnish (EV) coating/steel and epoxy glass flake (EGF) coating/steel systems were measured under ordinary pressure and marine hydrostatic pressure. The understanding of coating failure due to the degradation of wet adhesion has been discussed. GST was used to establish the grey models GM (1, 1) of wet adhesion, which are new in the corrosion field and valuable to be reported. Eventually, a lifetime prediction formula was developed based on the results above.

## 2. Results

### 2.1. Adhesion Results of the Coating/Steel Systems

**Figure 1:**
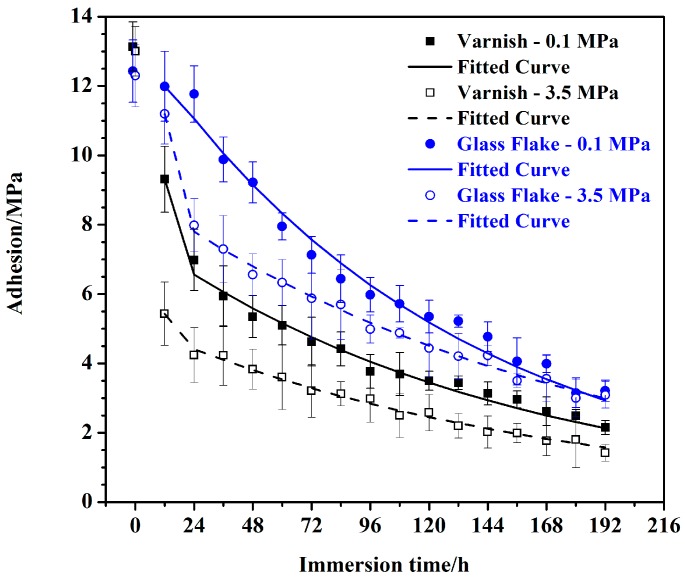
Changes of adhesion of the coating/steel systems under ordinary pressure (0.1 MPa) and hydrostatic pressure (3.5 MPa) (scatters: experimental data, solid and dash lines: fitting results of the grey models).

**Figure 2:**
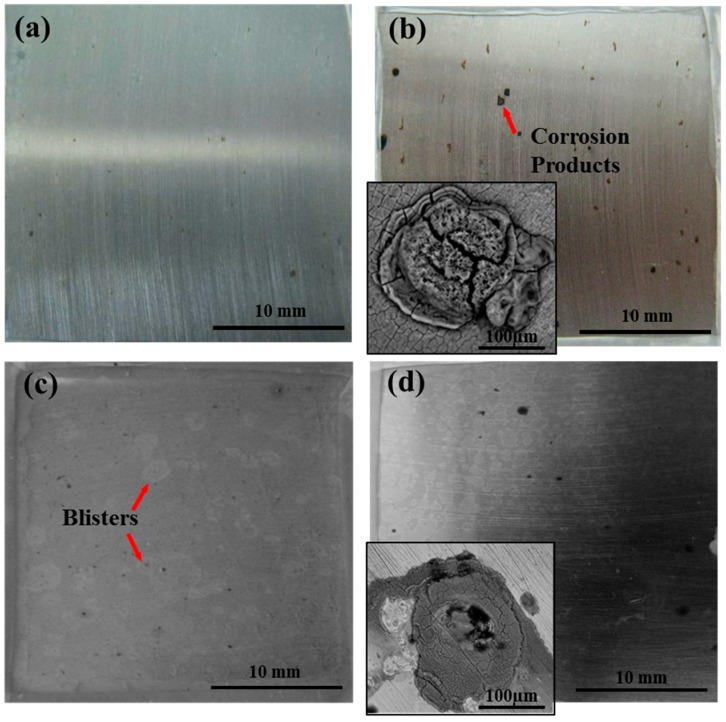
Macroscopic morphology pictures of epoxy varnish (EV) coating/steel under different pressure conditions: under ordinary pressure (**a**) after immersion 192 h and (**b**) after 720 h (30 d); under hydrostatic pressure (**c**) after 192 h and (**d**) after 720 h (30 d). The corresponding microscopic corrosion morphologies after 720 h under two conditions are shown in (**b**,**d**), respectively.

The changes of adhesion of EV coating/steel and EGF coating/steel under two environments are shown in Figure 1. The scatter plots represent the experimental data and the lines represent the fitting results of grey models, which will be discussed in the following sections. The average values of dry adhesion of EV coating and EGF coating are 13.14 MPa and 12.43 MPa, respectively, and the latter is a little lower, which may due to the addition of glass flake. However, the dry adhesion will change to be wet adhesion after immersion in the water. Referring to Figure 1, the declines in wet adhesion of the coatings under different pressures can be found during the entire test period. For the EV coating, wet adhesion under two pressure conditions decreased dramatically during the initial period; in particular, the degradation under hydrostatic pressure was greater than that under ordinary pressure. When the immersion time was 192 h, the wet adhesion under ordinary pressure and under hydrostatic pressure declined to a very low level, 2.15 MPa and 1.42 MPa, respectively. Several visible corrosion spots appeared on the substrate of the sample under ordinary pressure (Figure 2a). However, there mainly were obvious blisters for the sample under hydrostatic pressure (Figure 2c) at the same time, indicating that such poor wet adhesion could not ensure the protective performance of the organic coating. For the purpose of comparison, the macro morphology of samples under ordinary pressure after immersion 720 h is shown in Figure 2b, and a large amount of macroscopic corrosion products could be observed. However, blistering was the main cause for coating degradation rather than corrosion under hydrostatic pressure (Figure 2d). Microscopic corrosion morphologies showed that the corrosion was cystiform under ordinary pressure (inset in Figure 2b), while, under hydrostatic pressure, corrosion was observed in areas under the blisters (inset in Figure 2d). Consequently, different corrosion states were obtained under different pressure conditions.

**Figure 3:**
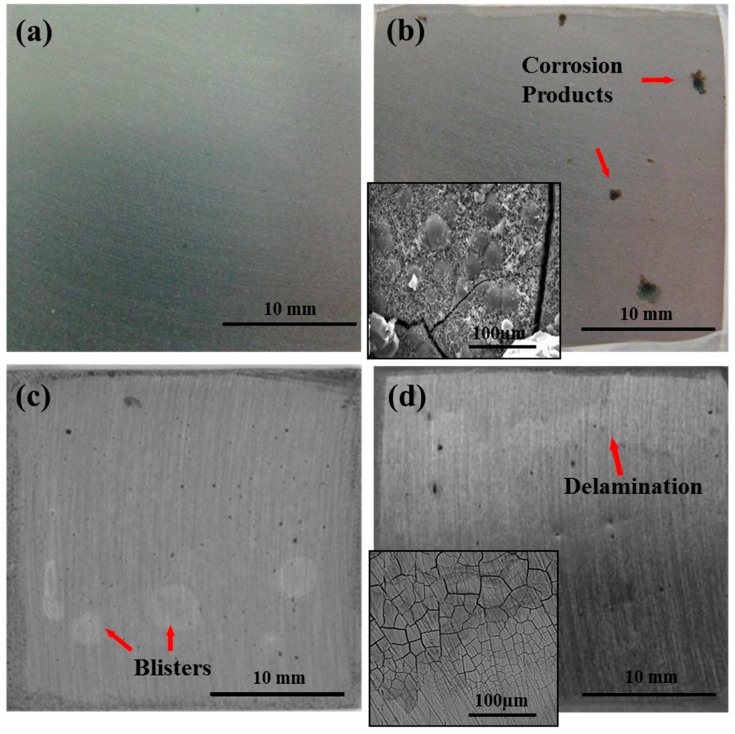
Macroscopic morphology pictures of epoxy glass flake (EGF) coating/steel under different pressure conditions: under ordinary pressure (**a**) after immersion 192 h and (**b**) after 720 h (30 d); under hydrostatic pressure (**c**) after 192 h and (**d**) after 720 h (30 d). The corresponding microscopic corrosion morphologies after 720 h under two conditions are shown in (**b**,**d**), respectively.

The variations on EGF coating/steel followed a similar changing trend. The wet adhesion under hydrostatic pressure degraded greater than that under ordinary pressure. After serving 192 h, the values under ordinary pressure and under hydrostatic pressure declined to 3.21 MPa and 3.09 MPa, respectively. Macro morphology in Figure 3a,c (serving 192 h) also indicated that the coating degradation under hydrostatic pressure was more severe for identical duration, with visible blisters and corrosion pitting. Finally, the failure states under ordinary pressure and hydrostatic pressure were mainly related to corrosion products (Figure 3b) and blisters (Figure 3d), respectively. 

**Figure 4:**
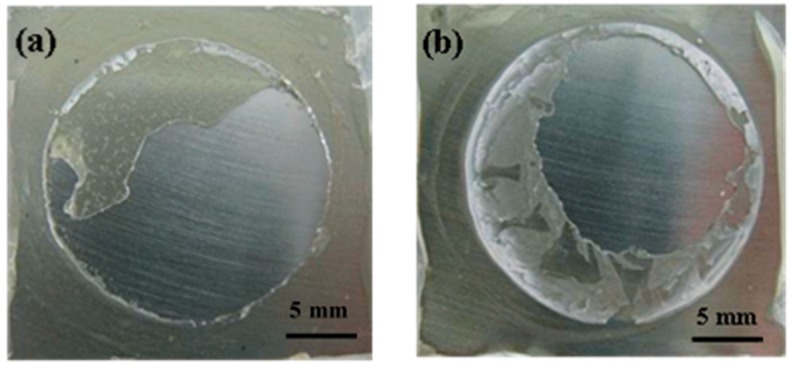
After pull off tests, the coatings have been released from substrates. The surface of substrates can been observed clearly. Macroscopic fracture morphology pictures of two coating/steel systems before immersion: (**a**) EV coating/steel; (**b**) EGF coating/steel.

**Figure 5:**
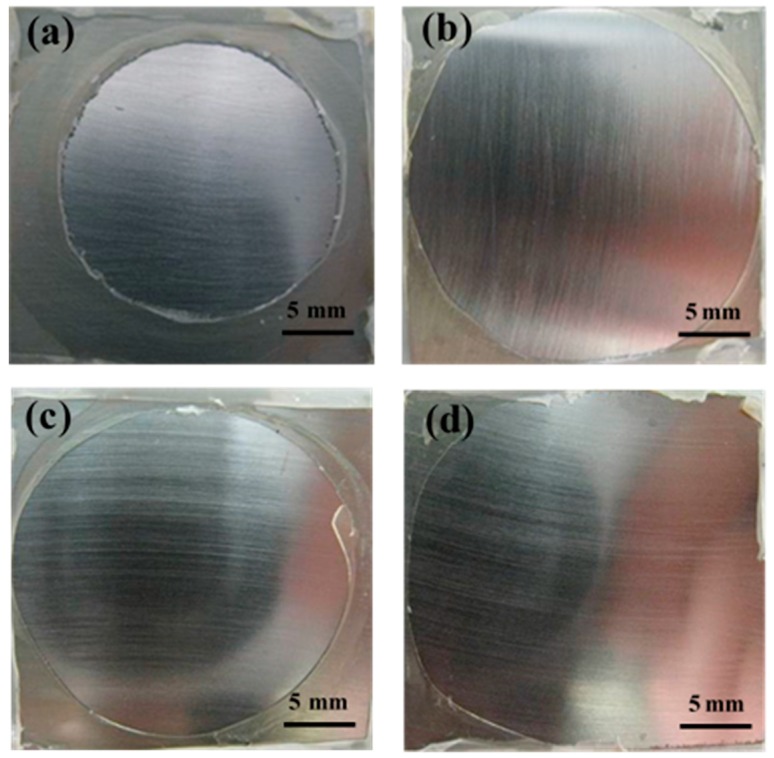
Macroscopic fracture morphology pictures of EV coating/steel under two pressure conditions at different immersion times: (**a**) after immersion 48 h under ordinary pressure; (**b**) 192 h under ordinary pressure; (**c**) 48 h under hydrostatic pressure and (**d**) 192 h under hydrostatic pressure.

**Figure 6:**
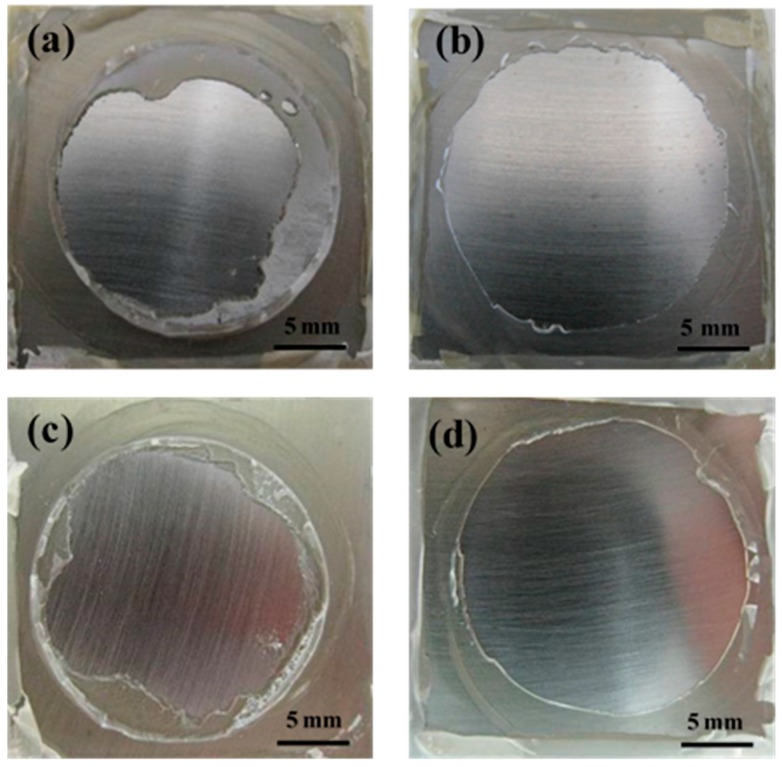
Macroscopic fracture morphology pictures of EGF coating/steel under two pressure conditions at different immersion times: (**a**) after immersion 48 h under ordinary pressure; (**b**) 192 h under ordinary pressure; (**c**) 48 h under hydrostatic pressure and (**d**) 192 h under hydrostatic pressure.

From the analysis above, the coating under hydrostatic pressure had a rapid loss of wet adhesion. The failure state was mainly related to the blisters, and dramatic adhesion degradation occurred before obvious corrosion of the substrate, which is different from that under ordinary pressure. In addition, EGF coating had a better protective performance than EV coating in the same condition, which was attributed to the barrier properties of glass flakes [9]. The changes in glass transition temperatures of the coatings have proved this (Table 1). In addition, comparisons of the fracture area of coatings after pull off tests were made to support the results above. In Figure 4, both coatings before immersion had a mixed adhesive/cohesive fracture form. In other words, the adhesion of coating/steel interface in some areas is even stronger than the coating structure, so that the coating body first fractured when a pull off test was performed, implying that both coatings have a very high dry adhesion. Then, the fracture form of the EV coating turned into a complete adhesive fracture (48 h, Figure 5a) followed by the failure fracture (192 h, Figure 5b) under ordinary pressure. By contrast, the EV coating under hydrostatic pressure had bigger exposed areas at each time period (Figure 5c,d). The EGF coating developed from mixed fracture (48 h, Figure 6a) to complete adhesive fracture (192 h, Figure 6b), and the coating under hydrostatic pressure had bigger exposed areas (Figure 6c,d). The photos of fracture surface suggest that the coatings under hydrostatic pressure have a faster interface delamination, and the glass flakes can allay the reduction of wet adhesion.

From the results above, the failure mechanisms of the coating can be demonstrated. For the coating under ordinary pressure, inevitable permeation of the water gradually causes coating degradation and substrate corrosion. However, the failure behaviour of coating is mainly related to blister rather than corrosion under high hydrostatic pressure. This is because the water diffusion is accelerated by high hydrostatic pressure. More water is accumulated at the coating/steel interface, resulting in the presence of many small blisters. Therefore, the adhesion decreases rapidly. As the immersion continues, the blisters would connect to each other. A water film with corrosion products forms on the interface (Figure 3d), at which time the coating fails eventually. 

### 2.2. Establishment of the GM (1, 1) Models by GST

In GST, the white system is the system in which the information is completely defined. The black system is the system in which the information is undefined. The grey system is the system in which some information is defined and some undefined. The grey prediction method of GST is making the most of the available information, to predict the future trends of an uncertain system from only a limited amount of data [21]. The GM (*α*, *β*) model is the core of grey prediction, where *α* is the order of the differential equation and *β* is the number of the variables. 

The GM (1, 1) model, which is composed of a differential equation of first order with one variable, is the most widely used one among different grey prediction models due to its computational efficiency [31]. In this work, GM (1, 1) was used to build the mathematical models on wet adhesion and conduct a further analysis of lifetime prediction of the coatings. In order to assess the precisions of the models, the first 12 groups of the original data were used to make the establishment, and the last four groups were stayed to examine the accuracy of the models. Taking the EV coating/steel system under 0.1 MPa, for example, the establishment of GM (1, 1) is shown as follows.

Defining a sequence $X^{\left(0\right)}$ that denotes the wet adhesion of a coating/steel system at different immersion times, and the initial sequence is:

(1) $$\{X^{\left(0\right)}\}=\{{X^{\left(0\right)}}_{\left(1\right)},{X^{\left(0\right)}}_{\left(2\right)},{X^{\left(0\right)}}_{\left(3\right)},...,{X^{\left(0\right)}}_{\left(n\right)}\}.$$

The corresponding time sequence is:

(2) $$\{t\}=\{t_{1},t_{2},t_{3},...,t_{n}\},$$

where $X^{\left(0\right)}$ is the wet adhesion (non-negative value), *t* is the immersion time, and *n* is the sample size of the data. Therefore, the sequence of the EV coating/steel system under 0.1 MPa is:

$$\{X^{\left(0\right)}\}=\{9.31,6.98,\;5.94,\;5.35,\;5.10,\;4.63,\;4.42,\;3.77,\;3.69,\;3.50,\;3.44,\;3.13\}.$$

The establishment of the corresponding grey model requires initial data with equal time intervals, and its specific time sequence is:

{t}={12, 24, 36, 48, 60, 72, 84, 96, 108, 120, 132, 144} .

The generation treatment of initial data, such as Accumulating Generation Operator (AGO) or Inverse AGO, is the premise of the establishment of the grey model, which can smooth the randomness and strengthen the regularity of the sequence. Here, the grey sequence generation is performed by AGO, and the following monotonically increasing sequence $X^{\left(1\right)}$ is obtained:

(3) $$\{X^{\left(1\right)}\}=\{{X^{\left(1\right)}}_{\left(1\right)},{X^{\left(1\right)}}_{\left(2\right)},{X^{\left(1\right)}}_{\left(3\right)},...,{X^{\left(1\right)}}_{\left(n\right)}\},$$

where

(4) $${X^{\left(1\right)}}_{\left(k\right)}=\sum_{i=1}^{k}{X^{\left(0\right)}}_{\left(i\right)},\left(k=1,2,3,...,n\right).$$

The generated mean sequence $Z^{\left(1\right)}$ of $X^{\left(1\right)}$ is defined as:

(5) $$\{Z^{\left(1\right)}\}=\{{Z^{\left(1\right)}}_{\left(2\right)},{Z^{\left(1\right)}}_{\left(3\right)},...,{Z^{\left(1\right)}}_{\left(n\right)}\},$$

where

(6) $${Z^{\left(1\right)}}_{\left(k\right)}=\frac{1}{2}[{X^{\left(1\right)}}_{\left(k\right)}+{X^{\left(1\right)}}_{\left(k-1\right)}],\left(k=2,3,...,n\right).$$

The AGO sequence $X^{\left(1\right)}$ and the generated mean sequence $Z^{\left(1\right)}$ of the EV coating/steel system under 0.1 MPa are calculated according to Equations (4) and (6), respectively. Then, the next step involves the building and solving of grey differential equation. GM (1, 1) is the first order differential equation model, and the form of equation is:

(7) $$\frac{dX}{dt}+aX=u.$$

The least square estimate sequence of the grey differential equation is defined as follows [31]:

(8) $${X^{\left(0\right)}}_{\left(k\right)}+a{Z^{\left(1\right)}}_{\left(k\right)}=u.$$

Then, the GM (1, 1) whitening differential equation of ${X^{\left(1\right)}}_{\left(k\right)}$ is therefore:

(9) $$\frac{d{X^{\left(1\right)}}_{\left(k\right)}}{dt}+a{X^{\left(1\right)}}_{\left(k\right)}=u.$$

In the above,

(10) $${\left[a,u\right]}^{T}={\left(B^{T}B\right)}^{-1}B^{T}Y_{n},$$

where

(11) $$B=[\begin{array}{l} -{Z^{\left(1\right)}}_{\left(2\right)}\\ -{Z^{\left(1\right)}}_{\left(3\right)}\\ .\\ .\\ .\\ -{Z^{\left(1\right)}}_{\left(n\right)} \end{array}\begin{array}{l} \;1\\ \;1\\ .\\ .\\ .\\ \;1 \end{array}],$$

(12) $$Y_{n}={\left[{X^{\left(0\right)}}_{\left(2\right)},{X^{\left(0\right)}}_{\left(3\right)},...,{X^{\left(0\right)}}_{\left(n\right)}\right]}^{T}.$$

By solving Equations (10)–(12) based on the data above, the parameters *a* and *u* of the EV coating/steel system under 0.1 MPa were 0.0804 and 7.5778, respectively.

According to Equation (9), the solution of ${X^{\left(1\right)}}_{\left(k\right)}$ at time *k* is:

(13) $${X_{p}^{\left(1\right)}}_{\left(k\right)}=[{X^{\left(0\right)}}_{\left(1\right)}-\frac{u}{a}]e^{-a(k-1)}+\frac{u}{a},\left(k=2,3,...,n\right).$$

In the above, *p* denotes the predicted value. Then, to obtain the predicted value of the primitive data at time *k*, the Inverse Accumulating Generation Operator (IAGO) is used to establish the following GM (1, 1):

(14) $${X_{p}^{\left(0\right)}}_{\left(k\right)}=[{X^{\left(0\right)}}_{\left(1\right)}-\frac{u}{a}]e^{-a(k-1)}(1-e^{a}),\left(k=2,3,...,n\right),$$

for 

(15) $$t=t_{1}+N(k-1).$$

Replacing the parameter *k* in Equation (14) with *t*, the following equation can then be obtained:

(16) $${X_{p}^{\left(0\right)}}_{\left(t\right)}=[{X^{\left(0\right)}}_{\left(1\right)}-\frac{u}{a}]e^{-a(\frac{t-t_{1}}{N})}(1-e^{a}),\left(t\geq t_{1}+N\right),$$

where *t*_1_ is the initial time of the time sequence *t*, and *N* is the interval of the arithmetic series. Equations (14) and (16) are grey models GM (1, 1) based on GST with different expressions for the wet adhesion of the organic coating/steel system. The same procedures were adopted for EV coating under 3.5 MPa and EGF coating under two pressures. Finally, the parameter values and the grey model GM (1, 1) formulas by Equation (16) are summarized in Table 2.

Figure 1 shows the fitting results of the established grey models (the values of dry adhesion have been ruled out). It can be seen that the calculated values of GM (1, 1) models are in good agreement with the experimental data, and the fitted curves reflect the changing trend of wet adhesion with immersion time very well.

## 3. Discussion

### 3.1. Precision Assessments of the Models

In this section, three evaluation standards for GST were used, including the relative error (RE), the average relative error (ARE) and the posterior error examination (PEE), in order to assess the reliability and accuracy of the proposed models.

The RE and ARE are defined as follows:

(17) $$\mathrm{RE}=\frac{{X^{\left(0\right)}}_{\left(k\right)}-{X_{p}^{\left(0\right)}}_{\left(k\right)}}{{X^{\left(0\right)}}_{\left(k\right)}}\times 100\%,$$

(18) $$\mathrm{ARE}=\frac{1}{n}\sum_{k=2}^{n}\frac{\left|{X^{\left(0\right)}}_{\left(k\right)}-{X_{p}^{\left(0\right)}}_{\left(k\right)}\right|}{{X^{\left(0\right)}}_{\left(k\right)}}\times 100\%,$$

where $X_{p}^{\left(0\right)}$ represents the calculated value of the model. According to Equation (17), the REs of GM (1, 1) for both coating systems under different pressures are obtained and shown in Table 3. The majority of the data are fitted well, which indicates that the models are acceptable.

**Figure 7:**
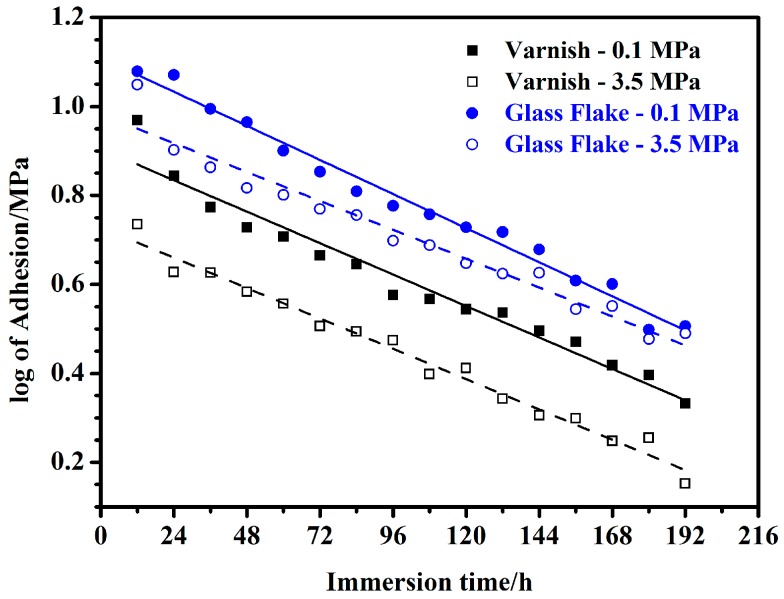
Log of adhesion-*t* fitted curves for the coatings under ordinary pressure and hydrostatic pressure.

For comparison, linear fitting for the log of adhesion with immersion time was made. Figure 7 shows the fitted curves for the coatings under ordinary pressure and hydrostatic pressure. It seems that the fitting results are also acceptable; however, the AREs of the log-linear fitting were compared with those of grey models (Table 4), which demonstrate that grey models have higher precision in general, especially for the data under hydrostatic pressure. Therefore, it is believed that GM (1, 1) is suitable for modeling the wet adhesion of coatings in our study.

In order to test the precisions further, the posterior error examination (PEE) method was used as follows:

$\varepsilon^{\left(0\right)}$ denotes the error residuals:

(19) $${\varepsilon^{\left(0\right)}}_{\left(k\right)}={X^{\left(0\right)}}_{\left(k\right)}-{X_{p}^{\left(0\right)}}_{\left(k\right)},\;\left(k=1,2,3,...,n\right).$$

Then, the variances of the initial sequence $X^{\left(0\right)}$ and the error residuals sequence $\varepsilon^{\left(0\right)}$ are:

(20) $${s_{1}}^{2}=\frac{1}{n}\sum_{k=1}^{n}[{X^{\left(0\right)}}_{\left(k\right)}-{\bar{X}}^{\left(0\right)}{]}^{2},$$

(21) $${s_{2}}^{2}=\frac{1}{n}\sum_{k=1}^{n}[{\varepsilon^{\left(0\right)}}_{\left(k\right)}-{\bar{\varepsilon }}^{\left(0\right)}{]}^{2},$$

where

(22) $${\bar{X}}^{\left(0\right)}=\frac{1}{n}\sum_{k=1}^{n}{X^{\left(0\right)}}_{\left(k\right)},$$

(23) $${\bar{\varepsilon }}^{\left(0\right)}=\frac{1}{n}\sum_{k=1}^{n}{\varepsilon^{\left(0\right)}}_{\left(k\right)}.$$

Consequently, the mean square error ratio *C* and the small error probability *p* are defined as:

(24) $$C=\frac{s_{2}}{s_{1}},$$

(25) $$p=\frac{1}{n}P,$$

where *P* is the number of the primitive data which meets $\left|{\varepsilon^{\left(0\right)}}_{\left(k\right)}-{\bar{\varepsilon }}^{\left(0\right)}\right|<0.6745s_{1}$.

The posterior error indexes *C* and *p* are used to assess the accuracy grades by comparing them with the accuracy standard of posteriori error (Table 5). The smaller *C* is and the larger *p* is, the more precise the models are. According to Equations (19)–(25), *C* and *p* for each system are: *C* = 0.11, *p* = 1 for EV coating under 0.1 MPa; *C* = 0.12, *p* = 1 for EV coating under 3.5 MPa; *C* = 0.15, *p* = 1 for EGF coating under 0.1 MPa; *C* = 0.10, *p* = 1 for EGF coating under 3.5 MPa. Comparing the *C* and *p* with the standard in Table 4, the accuracy grades of the proposed GM (1, 1) models for both coatings reach the “first class” precision, implying that the established grey models for wet adhesion of the coatings have high accuracy, which are consistent with the results of the precision assessments above.

### 3.2. Lifetime Prediction Based on GM (1, 1)

Based on our previous analysis, the wet adhesion is a significant factor determining the lifetime of the organic coating/steel system, especially the coatings under hydrostatic pressure. Consequently, it is necessary to make the predictions based on wet adhesion, and the proposed models will provide further accurate information for the study on lifetime of organic coatings. 

According to Equation (16), the relationship between wet adhesion and immersion time is obtained, and then the lifetime prediction formula can be derived:

(26) $$t_{\infty }=\frac{N}{a}\ln\frac{\left[{X^{\left(0\right)}}_{\left(1\right)}-\frac{u}{a}](1-e^{a}\right)}{W}+t_{1},$$

where *t*_∞_ is the theoretical lifetime of the coating, and *W* is defined as the critical value of wet adhesion.

According to Equation (26), it is easy to calculate the theoretical lifetime of the coatings once the critical wet adhesion is defined. Using the parameters from GM (1, 1), two critical values of wet adhesion were defined in this study: 1.1 MPa and 0.5 MPa. The predicted values from GM (1, 1) and log-linear fitting for EV coating/steel were compared with the experimental data (Table 6). It can be seen that the predicted results of grey model more approach the experiment results, illustrating that the forecasting formula obtained by GST has a high dependability and can be used to make precise predictions.

## 4. Materials and Methods 

### 4.1. Sample Preparation

**Figure 8:**
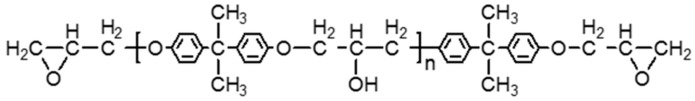
The molecular formula of E44 epoxy resin (bisphenol A).

The substrate was hot-rolled steel with composition (in wt.%): 4.67 Ni, 0.60 Cr, 0.46 Mo, 0.065 V, 0.54 Mn, 0.076 C, and Fe balance. The steel plate was cut into pieces with dimensions of 30 mm × 30 mm × 2 mm, and then was ground to #600 SiC paper and dewatered with ethanol. Two paints were brushed on the steel sheets, forming the EV coating and the EGF coating. The EV coating, consisting of E-44 resin (bisphenol A, Wuxi Resin Factory, Wuxi, China) as the binder (the molecular formula is shown in Figure 8), polyamide (TY-650, Tianjin Yanhai Chemical Co., Ltd., Tianjin, China) as the hardening agent and dimethylbenzene as the solvent, was mixed in the weight proportions of 1:0.8:0.3 for stoichiometric reaction. No adhesion promoter or other additive was used. The EGF coating, consisting of the same binder, hardening agent and solvent with glass flake as the pigment, was mixed in the weight ratio of 1:0.8:0.3:0.3. A commercial lamellar glass flake (thickness: 2–5 μm, diameter: 30–40 μm, Wen’an Huaxing Glass Flake Factory, Langfang, China) was used to make the single component pigmented coating system. Both coatings were stirred by a commercial magnetic stirrer machine for 2 h to mix sufficiently and then kept stationary for 0.5 h to ripen enough. Then, the coatings were brushed on the steel substrate and cured in an oven with the following conditions: 40 °C for 4 h, 60 °C for 20 h, and room temperature (25 °C, 30% relative humidity) for one week.

The coating thickness was measured by a hand-held electronic gauge (PosiTector 6000, Defelsko, New York City, NY, USA) according to ISO 2808 standard procedures [32], and an average thickness of 200 ± 10 µm was obtained. The edges of the specimens were sealed by a mixture of wax and colophony (volume ratio 1:1) to prevent edge effects that may have an influence on the test results.

### 4.2. Experimental Setup

**Figure 9:**
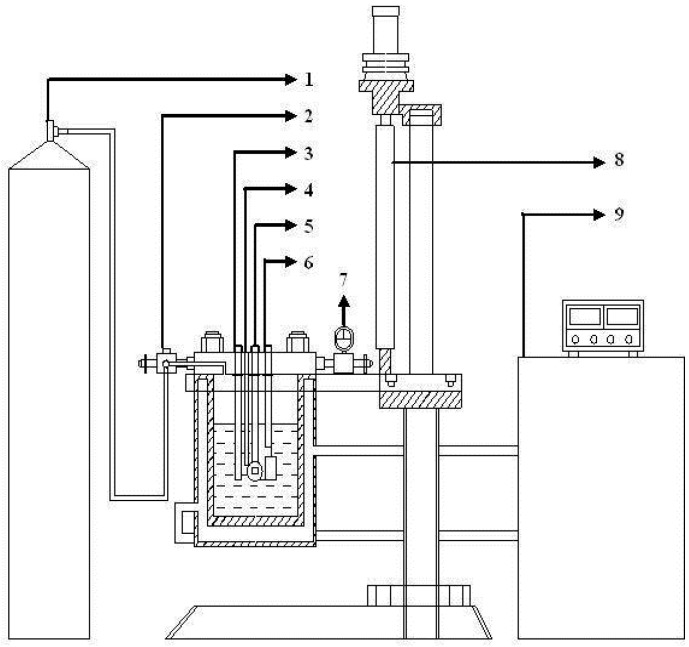
Schematic diagram of the deep ocean simulation device: (1) nitrogen cylinder; (2) valve; (3) solid reference electrode; (4) thermocouple; (5) working electrode; (6) counter electrode; (7) pressure meter; (8) automatic elevator and (9) temperature controller.

The immersion experiments under 0.1 MPa were conducted in ordinary pressure at room temperature. All experiments under 3.5 MPa were carried out in a specially designed deep ocean simulation system (as shown in Figure 9). The system included an autoclave, the hydrostatic system, pneumatic system, thermostatic system and monitoring system. The high hydrostatic pressure was obtained by pushing nitrogen into the autoclave, which was controlled by a pressure valve. Other details of the setup have been reported in [9]. The solution of 3.5 wt.% NaCl was prepared with analytical grade NaCl and distilled water. According to Henry’s Law, the concentration of dissolved oxygen remained constant during the pressurizing process [33].

### 4.3. Wet Adhesion Test

**Figure 10:**
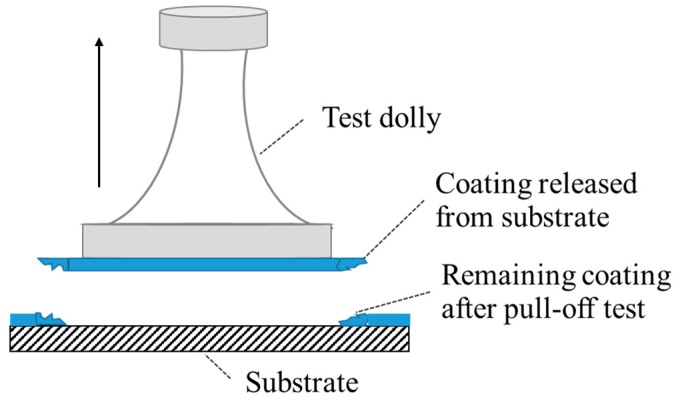
Schematic diagram of the pull off test.

Pull-off tests were used to detect the wet adhesion of organic coating/steel after different immersion times according to ASTM D4541-02 [34]. A Positest Pull-Off Adhesion Tester was used for getting the values of the adhesion. The pull off tests were done using the coated steel sheet sample. When immersion was finished, an Al dolly was pasted onto the coating surface. The remaining water on the surface of the coatings was removed swiftly by filter paper before the use of an adhesive that bonds the coating and the dolly. The sets were placed in an environment with a temperature between 30–40 °C for 3 h to make sure that the adhesive was cured completely and would not fail in the test. Then, the coating attached to the dolly was released from the substrate, leaving the remaining coating around the fracture (see Figure 10). After the pull off tests, the coatings were released from substrates. The surface of substrates can been observed clearly. Additionally, six parallel samples were tested and the final result was the average from these six sets. 

## 5. Conclusions

The rapid loss of wet adhesion is the immediate cause for coating failure under high hydrostatic pressure and thus the key factor controlling coating lifetime. The grey models GM (1, 1) based on wet adhesion were established firstly, which can well reflect the trend of wet adhesion with immersion time. Then, a lifetime prediction formula was developed by GM (1, 1), and the predicted results were in good agreement with the experimental data. The precision assessments illustrated that GST is an appropriate method for establishing a mathematical model of wet adhesion. The grey prediction model has good dependability and can make a precise forecasting of the lifetime of the coatings. Furthermore, the correctness of the grey model should be further confirmed in field applications, and the comparison between the experimental results and the field test results will be made in additional study.

## Figures and Tables

**Table 1:** The glass transition temperatures of the coatings before and after immersion.

Tg/°C	Before Immersion	720 h–0.1 MPa	720 h–3.5 MPa
Epoxy varnish coating	84	72	63
Epoxy glass flake coating	97	88	82

**Table 2:** The parameters calculated by grey system theory (GST) and the grey model (GM) (1, 1) formulas of the coatings under ordinary pressure (0.1 MPa) and hydrostatic pressure (3.5 MPa).

System	*a*	*u*	GM (1, 1) Formula
EV coating-0.1 MPa	0.0804	7.5778	7.11exp[−6.7 × 10^−3^(*t* − 12)]
EV coating-3.5 MPa	0.0734	4.9708	4.74exp[−6.1 × 10^−3^(*t* − 12)]
EGF coating-0.1 MPa	0.0948	12.7330	12.16exp[−7.9 × 10^−3^(*t* − 12)]
EGF coating-3.5 MPa	0.0685	8.8367	8.35exp[−5.7 × 10^−3^(*t* − 12)]

**Table 3:** The relative errors of the established GM (1, 1) grey models for the coatings under different pressures.

Immersion Time/h	EV-0.1 MPa	EV-3.5 MPa	EGF-0.1 MPa	EGF-3.5 MPa
12	0.00	0.00	0.00	0.00
24	6.02	−4.01	6.42	2.31
36	−2.02	3.07	−1.79	0.27
48	−4.49	0.52	0.77	−3.53
60	−1.18	1.67	−4.56	−0.31
72	−2.81	−2.49	−5.81	−0.84
84	0.68	2.24	−6.53	2.89
96	−7.43	4.70	−4.47	−3.48
108	−1.36	−5.6	0.35	1.04
120	1.43	5.04	3.28	−1.55
132	7.56	−3.64	10.83	0.00
144	6.07	−4.95	11.19	7.63
156	8.45	1.01	4.10	−4.63
168	4.58	−2.82	12.40	3.79
180	7.23	5.56	−2.48	−6.25
192	0.93	−11.27	9.56	3.34

**Table 4:** The average relative errors of GM (1, 1) grey models and log of adhesion-*t* fitting method.

System	GM (1, 1) Model	Log of Adhesion-*t* Fitting
EV-0.1 MPa	0.0389	0.0567
EV-3.5 MPa	0.0366	0.0405
EGF-0.1 MPa	0.0528	0.0435
EGF-3.5 MPa	0.0262	0.0516

**Table 5:** Accuracy standard of posteriori error [22].

Accuracy Grade	*C*	*p*
1st (Excellent)	≤0.35	≥0.95
2nd (Good)	0.35 < *C* ≤ 0.50	0.95 > *P* ≥ 0.80
3rd (Reasonable)	0.50 < *C* ≤ 0.65	0.80 > *P* ≥ 0.70
4th (Incorrect)	>0.65	<0.70

**Table 6:** The predicted values of two methods and the experimental data for EV coating/steel at different critical values of wet adhesion (1.1 MPa and 0.5 MPa).

System	EV-0.1 MPa		EV-3.5 MPa	
Critical wet adhesion/MPa	1.1	0.5	1.1	0.5
Actual lifetime/h	290	407	253	377
Predicted value of GM (1, 1)/h	291	408	251	380
Predicted value of log-linear fitting/h	293	410	242	362
